# Association of eosinophil‐derived neurotoxin levels with asthma control status in patients with aspirin‐exacerbated respiratory disease

**DOI:** 10.1002/clt2.12229

**Published:** 2023-03-06

**Authors:** Ga‐Young Ban, Eun‐Mi Yang, Young‐Min Ye, Hae‐Sim Park

**Affiliations:** ^1^ Department of Pulmonary, Allergy, and Critical Care Medicine Kangdong Sacred Heart Hospital Hallym University College of Medicine Seoul Korea; ^2^ Department of Allergy and Clinical Immunology, Allergy and Clinical Immunology Research Center Hallym University College of Medicine Seoul Korea; ^3^ Department of Allergy and Clinical Immunology Ajou University School of Medicine Suwon Korea

**Keywords:** aspirin‐exacerbated respiratory disease, asthma control, biomarker, eosinophils, eosinophil‐derived neurotoxin

## Abstract

**Background:**

The long‐term goals of asthma treatment are to achieve well control of symptoms and to minimize the future risk of asthma exacerbation. Identifying biomarkers for uncontrolled asthma is important for improving the asthma outcome. This study aimed to investigate the association of the levels of eosinophil‐derived neurotoxin (EDN) with asthma control status in specific asthma phenotype, aspirin‐exacerbated respiratory disease (AERD), and aspirin‐tolerant asthma (ATA).

**Methods:**

A total of 136 adult asthmatics, including 47 asthmatics with AERD and 89 asthmatics with ATA, were enrolled. Plasma, sputum, and urine were collected at enrollment and the levels of EDN were measured by the K‐EDN ELISA kit. Urinary leukotriene E4 (LTE_4_) level was measured using liquid chromatography–mass spectrometry (LC‐MS)/MS methods. Asthma control status was evaluated according to the GINA guideline, asthma control test and asthma control questionnaire scores.

**Results:**

In the total study subjects, sputum levels of EDN as well as of urine and plasma EDN showed significantly higher levels in patients with uncontrolled asthma than in those with well‐controlled or partly‐controlled asthma (ANOVA, *p* < 0.001); in patients with AERD, the sputum EDN levels showed significant correlations with ACT, ACQ, and AQLQ scores (*p* = 0.010, *r =* −0.536, *p* = 0.001, *r =* 0.665, and *p* < 0.001, *r =* −0.691, respectively), while no differences were noted in patients with ATA. Sputum EDN level was the only significant factor for ACT, ACQ, and AQLQ scores in patients with AERD (*p* = 0.001, *p* < 0.001, and *p* < 0.001, respectively) in the multivariate analysis adjusting for age, sex, peripheral eosinophil count, and urine LTE_4_. The ROC curve analysis demonstrated that sputum EDN can predict uncontrolled asthma with 80% sensitivity and 88.2% specificity for ACT ≤ 19 (area under the ROC curve [AUC] = 0.824, *p* = 0.019); 71.4% sensitivity and 86.7% specificity for ACQ ≥ 1.5 (AUC = 0.752, *p* = 0.049) only in AERD patients.

**Conclusion:**

The level of sputum EDN may be a potential biomarker for identifying the asthma control status in patients with AERD.

## 1 INTRODUCTION

Asthma is a heterogeneous disease which is characterized by chronic airway inflammation.^1^ Asthma can be classified as that with and without T_H_2 inflammation.^2^ T_H_2‐high asthma endotype typically shows eosinophilic inflammation, whereas T_H_2‐low asthma endotype is associated with the neutrophilic or paucigranulocytic inflammation.^3^ Eosinophilic inflammation induces airway remodeling and loss of asthma control, resulting in frequent asthma exacerbations (AE).^4^, ^5^

The long‐term goals of asthma treatment are to achieve well control of symptoms and to minimize the future risk of AE.^1^ Estimated annual frequencies of AEs per patient were reported to be 0.34–0.91 even in adult asthmatics having been treated with anti‐asthmatic medications.^6^ In a large asthma cohort study in Korea, about 28.6% of asthmatics experienced ≥1 AE and 8.5% experienced ≥3 AE within the first 2 years of treatment.^7^ Although the loss of asthma control is more frequent in patients with severe asthma (SA) than in those with mild to moderate asthma, it can occur regardless of asthma severity.^8^ Uncontrolled asthma (UA) is a critical factor for asthma‐associated morbidity and mortality and may increase health care cost of asthmatics and government public health agencies.^9^ Therefore, identifying the biomarkers for UA is important for improving asthma outcome.

To date, several studies have shown that T_H_2‐driven biomarkers, including peripheral blood eosinophil count (PEC), sputum eosinophil count (SEC), and fractional exhaled nitric oxide (FeNO) could predict poor asthma outcome.^10^, ^11^ In clinical practice, SEC measurement requires specific technique with facility and sometimes shows variable results influenced by current treatment. Assessment of FeNO is not strongly recommended for outcome measurement due to the inconsistent study results.^12^ Considering the consistency of the result and convenience of the measurement, PEC is the most frequently used biomarker; however, it may not always exactly reflect the degree of airway inflammation.^13^

Aspirin‐exacerbated respiratory disease (AERD) is characterized by asthma, chronic rhinosinusitis with nasal polyp, and hypersensitivity to nonsteroidal anti‐inflammatory drugs (NSAIDs)/aspirin. AERD represents a distinct endotype with dysregulation of arachidonic acid metabolism and upregulated T_H_2 inflammation.^14^ Overproduction of cysteinyl leukotrienes which potentially induce eosinophilic inflammation is the hallmark of AERD in pathogenic mechanism. It has been reported that asthmatics with AERD are most likely to have severe disease, which has a higher risk of UA, SA, and AE.^15^ Although urinary leukotriene E_4_ has been suggested as a biomarker for the diagnosis of AERD,^16^, ^17^ biomarkers for UA in AERD patients are lacking.

In the present study, we prospectively enrolled asthmatics with AERD and ATA that showed various asthma control status to evaluate the association of the levels of eosinophil‐derived neurotoxin (EDN) with clinical and inflammatory parameters as well as asthma control status.

## 2 METHODS

### 2.1 Study design and study population

A total of 136 patients with asthma were prospectively enrolled at Ajou University Hospital (Suwon, South Korea). Asthma was diagnosed according to the Global Initiative for Asthma guideline (GINA) 2022 by the allergy specialists.^1^ Asthmatics were classified into three groups according to their symptom control status: UA, partly‐controlled asthma (PA), and well‐controlled asthma (CA). Exclusion criteria for enrollment were as follows: (1) asthmatics who had been treated with biologics, including omalizumab, mepolizumab, reslizumab, and dupilumab within 130 days of enrollment; (2) current smokers or ex‐smokers who quit smoking within 30 days of enrollment; and (3) asthmatics whose controller medications were changed within 7 days of enrollment.

AERD was defined by a typical clinical history (recurrent exacerbation of upper or lower respiratory reactions after ingestion of NSAIDs/aspirin) and/or a positive response to the lysine‐aspirin bronchial provocation test (Lys‐ASA BPT). The Lys‐ASA BPT was performed with increasing doses of Lys‐ASA solution up to 300 mg/ml using the method previously reported.^18^ The Lys‐ASA BPT result was considered positive if forced expiratory volume in one second (FEV_1_)% was decreased by more than 20% after the challenge. ATA was defined when subjects showed negative results to the Lys‐ASA BPT or denied any upper or lower respiratory tract symptom changes after ingestion of NSAIDs/aspirin. Asthma control status was evaluated according to the GINA guideline,^1^ asthma control test (ACT), and asthma control questionnaire (ACQ‐6: ACQ mean of six individual item scores).^19^ UA was defined when ACT ≤ 19 or ACQ ≥ 1.5.^1^ SA was diagnosed according to the definition of international European respiratory society/American thoracic society guidelines.^20^ Eosinophilic asthma was defined as the PEC ≥ 300/μl.

### 2.2 Clinical data and sample collection

At the day of enrollment, peripheral venous blood, sputum, and urine samples were collected from the subjects between 8:00 a.m. and 11:00 a.m., when the patients had maintained on controller medications. Pulmonary function test, PC20 methacholine, FeNO measurement, and questionnaires survey using the ACT, ACQ‐6 (ACQ mean of six individual item scores), and asthma quality of life (AQLQ[S]) were performed on the same day of enrollment. Serum total immunoglobulin E level was measured using ImmunoCAP (ThermoFisher Scientific, Waltham, MA, USA). All subjects gave written informed consent at the time of enrollment, and the study was approved by the Institutional Review Board of Ajou University Hospital (AJIRB‐BMR‐SUR‐15‐498).

### 2.3 Measurement of EDN and LTE_4_

The samples of plasma, sputum, and urine were collected at enrollment and stored at −70°C. Levels of EDN in plasma, supernatant of sputum, and urine were measured using K‐EDN kit (SKIMS‐BIO Co., Seoul, Korea) as previously described.^21^ Serum and urine levels of leukotriene E4 (LTE_4_) were analyzed by liquid chromatography–tandem mass spectrometry. LTE_4_‐d5 (Cayman Chemical Company, Ann Arbor, MI, USA) was used as a deuterated internal standard. Chromatographic separations were performed using the Waters Acquity UPLC system (Waters) with a Hypersil GOLD column (2.1 × 100 mm, 1.9 μm: ThermoFisher Scientific, San Jose, CA, USA) on a concentration gradient. Data acquisition was performed using an API5500 triple quadrupole mass spectrometer (AB Sciex, Framingham, MA, USA) equipped with an Electrospray ionization source. For the quantitative determination of creatinine in urine samples, 10 μl of urine sample was applied to the Creatinine Parameter Assay Kit (R&D Systems, Minneapolis, MN, USA).

### 2.4 Statistical analysis

Student's *t* test, and Pearson's chi‐squared test were used for continuous and categorical variables, respectively. Analysis of variance (ANOVA) was performed for comparisons among the three groups. Multivariate logistic regression analysis was performed to determine significant factors affecting asthma control status. A receiver operating characteristic (ROC) curve analysis was performed to determine whether the EDN level help detect UA. All computations were performed using Statistical Package for the Social Sciences software, version 22.0 (IBM Corp., Armonk, NY, USA). GraphPad Prism 5.0 software (GraphPad Inc., San Diego, CA, USA) was used for the production of graphs.

## 3 RESULTS

### 3.1 Clinical characteristics of the study subjects

A total of 47 patients with AERD and 89 patients with ATA were enrolled. Table 1 shows the demographic data from the study subjects. Patients with AERD showed higher PEC than those with ATA (*p* = 0.013). The number of atopic patients were higher in ATA patients (*p* = 0.004). There were no other significant differences between patients with AERD and ATA.

**TABLE 1:** Demographic data of the study subjects.

Variables	AERD (*n* = 47)	ATA (*n* = 89)	*p* value
Age (year)	51.75 ± 11.85	49.36 ± 16.24	0.332
Sex (female)	33 (70.2%)	56 (62.9%)	0.395
Atopy (%)	18 (40.9%)	60 (67.4%)	0.004
Total IgE (KU/L)	246.23 ± 283.67	376.76 ± 461.35	0.088
Sputum eosinophil (%)	29.48 ± 35.67	23.60 ± 31.30	0.428
Blood eosinophil count (per μl)	393.62 ± 288.48	272.47 ± 206.33	0.013
FeNO (ppb)	39.94 ± 37.37	31.22 ± 28.48	0.171
FEV_1_ (% Pred)	90.00 ± 19.49	90.45 ± 16.75	0.889
FVC (% Pred)	91.72 ± 15.60	90.19 ± 14.62	0.574
FEV_1_/FVC	81.31 ± 9.02	83.93 ± 8.42	0.095
PC_20_ of methacholine (mg/ml)	5.19 ± 7.48	7.17 ± 8.43	0.231
Asthma control status^a^			
Well controlled	19 (40.4%)	52 (58.4%)	0.100
Partly controlled	22 (46.8%)	26 (29.2%)	
Uncontrolled	6 (12.8%)	11 (12.4%)	
ACT score	21.15 ± 3.11	20.40 ± 4.06	0.232
ACQ score	0.77 ± 0.83	1.05 ± 0.96	0.084
AQLQ score	5.40 ± 1.16	5.18 ± 1.21	0.318
Severe asthma	9 (19.1%)	15 (16.9%)	0.738
Sputum EDN (μg/μl)	1438.93 ± 1301.85	975.10 ± 765.86	0.109
Urine EDN (mg/dl cr)	6.40 ± 7.31	4.24 ± 3.61	0.068
Plasma EDN (ng/ml)	17.93 ± 14.13	14.78 ± 12.48	0.202

Abbreviations: ACT, asthma control test; ACQ, asthma control questionnaire; AERD, aspirin‐exacerbated respiratory disease; AQLQ, asthma quality of life questionnaire; ATA, aspirin tolerant asthma; EDN, eosinophil‐derived neurotoxin; FeNO, fractional exhaled nitric oxide; FEV_1_, forced expiratory volume in 1 s; FVC, forced vital capacity; IgE, immunoglobulin E; PC20, provocative concentration causing 20% fall in FEV_1_.

^a^ Defined by GINA guideline. Data were analyzed by student's *t*‐test and Pearson chi‐square.

### 3.2 Levels of sputum, urine, and plasma EDN according to asthma control status defined by the GINA guideline

**FIGURE 1:**
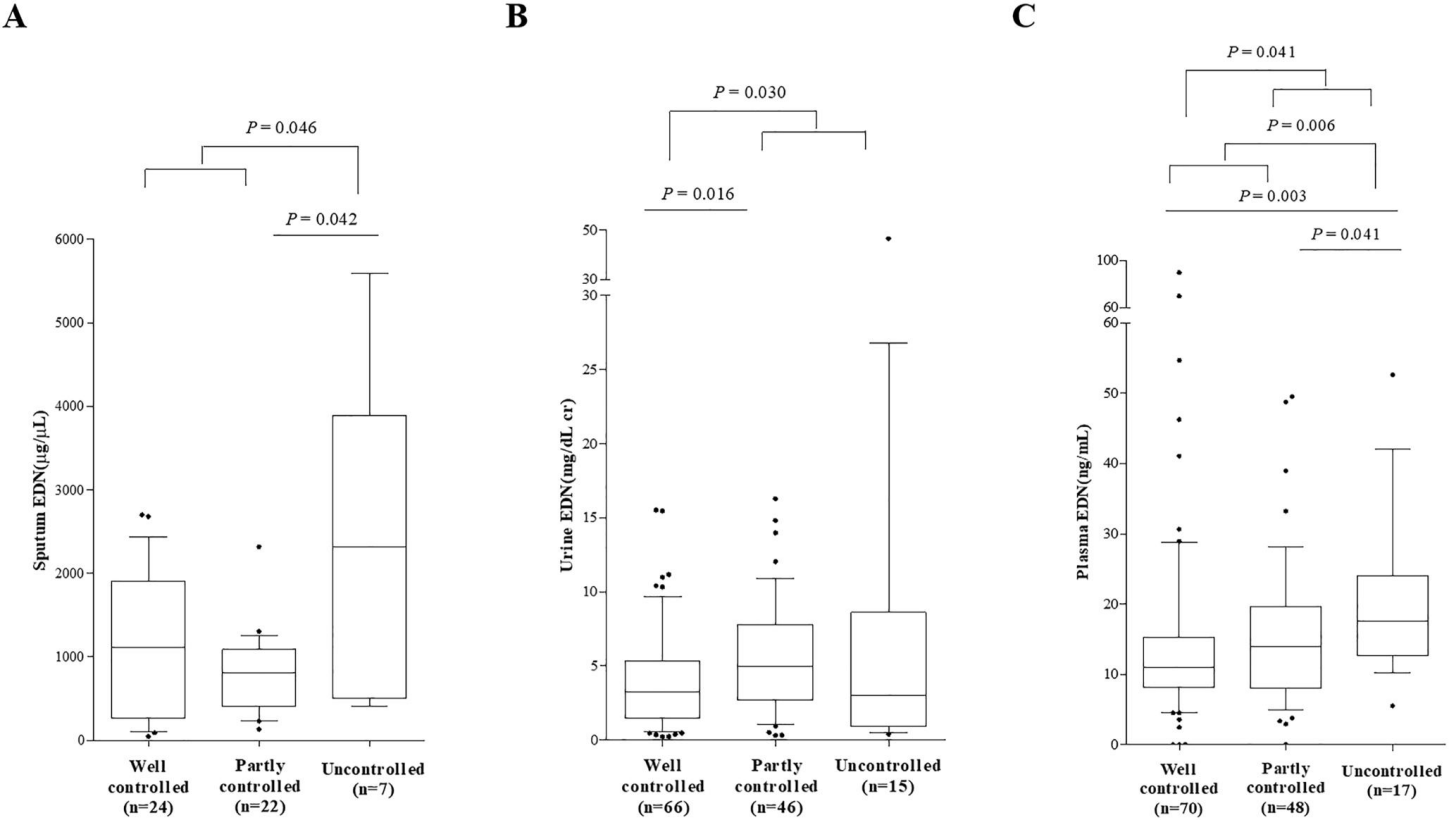
EDN levels in sputum (A), urine (B), and plasma (C) according to asthma control status in asthmatic subjects. Asthma control status was defined by the GINA guideline. EDN, eosinophil‐derived neurotoxin.

In the total number of study subjects, the sputum and urine levels of EDN showed significant difference among CA, PA, and UA groups defined by the GINA guideline (ANOVA, *p* = 0.002 and *p* = 0.049). The sputum and plasma levels of EDN were significantly higher in the UA group than in the others (*p* = 0.046 and *p* = 0.006). The urine and plasma levels of EDN were significantly lower in the CA group than in the others (*p* = 0.030 and *p* = 0.041) (Figure 1).

In patients with AERD, sputum levels of EDN showed significant difference among the CA, PA, and UA groups (ANOVA, *p* < 0.001), while no differences were noted in the urine and plasma levels of EDN. The levels of sputum EDN were significantly higher in AERD patients with UA than in those with CA or PA (*p* < 0.001 for all). Patients with ATA showed no differences in the levels of EDN according to asthma control status by the GINA guideline.

### 3.2 Correlation between EDN levels and asthma control questionnaire scores

**FIGURE 2:**
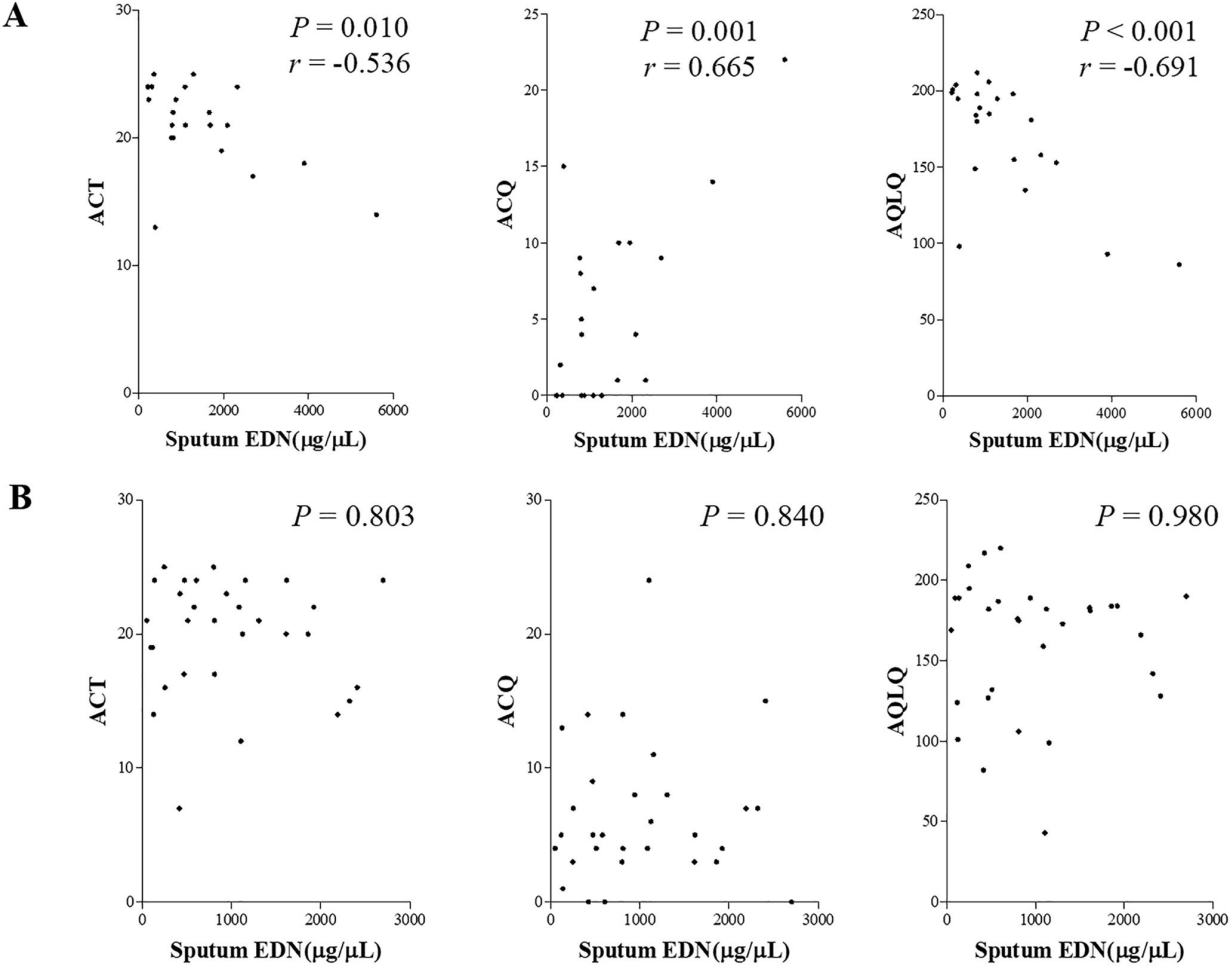
Correlations between sputum EDN level and asthma control status in patients with AERD (A) and those with ATA (B). ACT, asthma control test; ACQ, asthma control questionnaire; AQLQ, asthma quality of life questionnaire; AERD, aspirin‐exacerbated respiratory disease; ATA, aspirin tolerant asthma; EDN, eosinophil‐derived neurotoxin.

The sputum levels of EDN were significantly correlated with the ACQ and AQLQ scores in asthmatic subjects (*p* = 0.007, *r =* 0.369, and *p* = 0.028, *r =* −0.301, respectively). In patients with AERD, sputum EDN levels showed significant correlations with ACT, ACQ, and AQLQ scores (*p* = 0.010, *r =* −0.536; *p* = 0.001, *r* = 0.665; and *p* < 0.001, *r* = −0.691, respectively), while no correlations were found in patients with ATA (Figure 2). The urine and plasma levels of EDN showed no significant correlation with ACQs. The levels of PEC, SEC, or FeNO had no significant correlation with ACT, ACQ, or AQLQ scores in asthmatics with AERD, or ATA.

In subgroup analysis of eosinophilic asthma (AERD, *n* = 29; ATA, *n* = 38), sputum EDN levels showed significant correlations with ACT, ACQ, and AQLQ scores (*p* < 0.001 for all, *r =* 0.809, 0.866, and −0.925, respectively) in patients with AERD, while no correlations were found in patients with ATA.

### 3.3 Correlation between EDN levels and pulmonary function tests

The sputum EDN levels were negatively correlated with the predicted % value of FEV_1_ or FEV_1_/FVC (forced vital capacity) ratio in asthmatic subjects, especially in patients with AERD (*p* = 0.001, *r =* −0.646 and *p* = 0.008, *r =* −0.551, respectively), but not in those with ATA. In addition, urine, and plasma levels of EDN were significantly correlated with FEV_1_/FVC ratio in asthmatic subjects. However, ATA patients had no correlations between the levels of urine/plasma EDN and pulmonary function test results (Table 2).

**TABLE 2:** Correlations between the EDN levels and pulmonary function test results.

	Asthma (*n* = 136)			AERD (*n* = 47)			ATA (*n* = 89)		
Variable	Sputum EDN	Urine EDN	Plasma EDN	Sputum EDN	Urine EDN	Plasma EDN	Sputum EDN	Urine EDN	Plasma EDN
FEV_1_ (% pred)	*p* = 0.033 *r* = −0.295	*p* = 0.513	*p* = 0.083	*p* = 0.001 *r* = −0.646	*p* = 0.196	*p* = 0.054	*p* = 0.581	*p* = 0.217	*p* = 0.595
FVC (% pred)	*p* = 0.096	*p* = 0.613	*p* = 0.270	*p* = 0.002 *r* = −0.634	*p* = 0.541	*p* = 0.070	*p* = 0.358	*p* = 0.057	*p* = 0.986
FEV_1_/FVC	*p* = 0.024 *r* = −0.312	*p* = 0.008 *r* = −0.235	*p* = 0.012 *r* = −0.218	*p* = 0.008 *r* = −0.551	*p* = 0.085	*p* = 0.105	*p* = 0.752	*p* = 0.110	*p* = 0.091

Abbreviations: AERD, aspirin‐exacerbated respiratory disease; ATA, aspirin tolerant asthma; EDN, eosinophil‐derived neurotoxin; FEV_1_, forced expiratory volume in 1 s; FVC, forced vital capacity.

### 3.4 Correlations between EDN levels and inflammatory biomarkers

The EDN levels showed positive correlations with PEC (sputum EDN: *p* = 0.004, *r =* 0.390; urine EDN: *p* < 0.001, *r =* 0.307; plasma EDN: *p* < 0.001, *r =* 0.578, respectively), SEC (sputum EDN: *p* < 0.001, *r =* 0.581; urine EDN: *p* = 0.109; and plasma EDN: *p* = 0.040, *r =* 0.222, respectively), and FeNO values (sputum EDN, *p* = 0.457; urine EDN, *p* = 0.130; and plasma EDN, *p* = 0.049, *r =* 0.172, respectively) in asthmatic subjects.

LTE_4_ levels were also significantly correlated with EDN levels. The levels of serum LTE_4_ showed positive correlations with sputum/plasma levels of EDN (*p* = 0.011, *r =* 0.347, and *p* = 0.008, *r =* 0.226, respectively), and urine LTE_4_ levels showed significant correlations with all types of EDN (sputum EDN: *p* = 0.023, *r =* 0.311; urine EDN: *p* < 0.001, *r =* 0.362 and plasma EDN: *p* < 0.001, *r =* 0.388, respectively) in asthmatic subjects. Table 3 shows the correlation of the LTE_4_ and EDN levels in asthmatics with AERD and ATA patients.

**TABLE 3:** Correlations between the levels of LTE_4_ and EDN in patients with AERD or ATA.

	Asthma (*n* = 136)			AERD (*n* = 47)			ATA (*n* = 89)		
	Sputum EDN	Urine EDN	Plasma EDN	Sputum EDN	Urine EDN	Plasma EDN	Sputum EDN	Urine EDN	Plasma EDN
Serum LTE_4_	*p* = 0.011	*p* = 0.160	*p* = 0.008	*p* = 0.003	*p* = 0.368	*p* = 0.017	*p* = 0.505	*p* = 0.576	*p* = 0.419
	*r* = 0.347		*r* = 0.226	*r* = 0.605		*r* = 0.348			
Urine LTE_4_	*p* = 0.023	*p* < 0.001	*p* < 0.001	*p* = 0.218	*p* = 0.013	*p* = 0.002	*p* = 0.303	*p* = 0.039	*p* = 0.001
	*r* = 0.311	*r* = 0.362	*r* = 0.388		*r* = 0.369	*r* = 0.445		*r* = 0.228	*r* = 0.334

Abbreviations: AERD, aspirin‐exacerbated respiratory disease; ATA, aspirin tolerant asthma; EDN, eosinophil‐derived neurotoxin; LT, leukotriene.

### 3.5 Factors associated with asthma control status

In the univariate analysis, PEC and sputum EDN level were significant factors for UA defined by the GINA guideline (*p* = 0.042, Exp 〔B〕 = 1.002〔1.000068 − 1.004〕, *p* = 0.017, Exp 〔B〕 = 1.001〔1.0002 − 1.002〕, respectively) in asthmatic subjects. Sputum EDN level was the only significant factor for ACQ and AQLQ scores (*p* = 0.004, Exp 〔B〕 = 1.002〔1.001 − 1.003〕; *p* = 0.022, Exp 〔B〕 = 0.988〔0.979 − 0.998〕, respectively) in asthmatic subjects; for ACT, ACQ, and AQLQ scores (*p* = 0.003, Exp 〔B〕 = 0.999[0.998 − 0.999]; *p* < 0.001, Exp 〔B〕 = 1.003[1.002 − 1.002]; and *p* < 0.001, Exp 〔B〕 = 0.980〔0.971 − 0.989〕, respectively) in patients with AERD. Urine and plasma EDN levels showed no significant associations. The levels of PEC, SEC, urine LTE_4_, or FeNO were not a significant factor for ACT, ACQ, or AQLQ scores. All of these factors showed no statistical significances with asthma control status in patients with ATA.

In the multivariate analysis adjusting for age, sex, PEC, and urine LTE_4_, sputum EDN level was the only significant factor for UA defined by the GINA (*p* = 0.017, Exp 〔B〕 = 1.002〔1.000292 − 1.003〕) in asthmatic subjects. Table 4 shows the results of multivariate logistic regression analysis, whether the EDN levels are associated with ACQ scores after the adjustment of age, sex, PEC, and urine LTE_4_. Sputum EDN level was the significant factor for ACQ and AQLQ scores in asthmatic subjects (*p* = 0.004 and *p* = 0.022, respectively), and for ACT, ACQ, and AQLQ scores in patients with AERD (*p* = 0.001, *p* < 0.001, and *p* < 0.001, respectively), while no associations were observed in those with ATA.

**TABLE 4:** Factors affecting asthma control status in asthmatics.

		Asthma (*n* = 136)		AERD (*n* = 47)		ATA (*n* = 89)	
		*p* value	Exp (B)	*p* value	Exp (B)	*p* value	Exp (B)
ACT	Sputum EDN	0.139	0.999 (0.998 − 1.000)	0.001	0.998 (0.998 − 0.999)	0.892	1.000 (0.998 − 1.002)
	Urine EDN	0.115	1.112 (0.974 − 1.269)	0.105	1.120 (0.977 − 1.283)	0.546	1.079 (0.843 − 1.380)
	Plasma EDN	0.305	1.032 (0.972 − 1.097)	0.907	1.006 (0.915 − 1.105)	0.178	1.054 (0.976 − 1.137)
ACQ	Sputum EDN	0.004	1.002(1.001 − 1.004)	<0.001	1.004 (1.002 − 1.005)	0.790	1.000 (0.997 − 1.002)
	Urine EDN	0.419	0.923 (0.761 − 1.120)	0.367	0.904 (0.726 − 1.126)	0.942	0.987 (0.700 − 1.393)
	Plasma EDN	0.309	0.955 (0.873 − 1.044)	0.942	1.005 (0.867 − 1.166)	0.061	0.902 (0.809 − 1.005)
AQLQ	Sputum EDN	0.022	0.987 (0.976 − 0.998)	<0.001	0.978 (0.969 − 0.987)	0.707	1.004 (0.983 − 1.025)
	Urine EDN	0.316	1.982 (0.521 − 7.540)	0.549	1.626 (0.331 − 7.996)	0.369	2.934 (0.281 − 30.672)
	Plasma EDN	0.511	1.228 (0.666 − 2.264)	0.837	0.892 (0.302 − 2.635)	0.205	1.608 (0.772 − 3.349)

Abbreviations: ACT, asthma control test; ACQ, asthma control questionnaire; AQLQ, asthma quality of life questionnaire; AERD, aspirin‐exacerbated respiratory disease; ATA, aspirin tolerant asthma; EDN, eosinophil‐derived neurotoxin.

In subgroup analysis of eosinophilic asthma, sputum EDN levels were significantly associated with ACT, ACQ, and AQLQ scores in patients with AERD (*p* < 0.001 for all, Exp 〔B〕 = 0.998〔0.998 − 0.999〕, 1.004〔1.003 − 1.005〕, and 0.975〔0.971 − 0.980〕, respectively), while no associations were found in those with ATA.

The ROC curve analysis demonstrated that sputum EDN can predict UA with 80% sensitivity and 88.2% specificity for ACT ≤ 19 (area under the ROC curve [AUC] = 0.824, *p* = 0.019), 71.4% sensitivity and 86.7% specificity for ACQ ≥ 1.5 (AUC = 0.752, *p* = 0.049) in AERD patients, but not in ATA patients. The levels of PEC did not reach statistical significance for UA in ROC curve analysis.

## 4 DISCUSSION

This study evaluated the association of the sputum, urine, and plasma levels of EDN with asthma control status in patients with AERD and ATA. Even though all study subjects had maintained anti‐asthmatic medications, significant correlations were found between sputum EDN levels and asthma control status/lung function parameters in AERD patients, but not in ATA patients. Furthermore, sputum EDN was a significant factor associated with ACT, ACQ, and AQLQ scores in AERD patients even after adjustment for potential confounders such as PEC and urinary level of LTE_4_. Collectively, sputum EDN is a useful biomarker for detecting patients with UA, when monitoring patients with AERD.

Eosinophils contain potent granule proteins including EDN, eosinophil cationic protein (ECP), eosinophil peroxidase (EPO), and the major basic protein (MBP).^22^ EDN and ECP are released almost exclusively from eosinophils and induce tissue damage with dysfunction, mucus hypersecretion, as well as airway inflammation and remodeling. Recent studies reported that the level of serum EDN was higher in severe asthmatics than in nonsevere asthmatics,^23^ and in the uncontrolled asthmatics than in the controlled asthmatics, suggesting a close association between EDN and asthma severity or control status.^24^, ^25^ High EDN level at baseline was associated with persistent asthma (the persistence of nocturnal shortness of breath and chest tightness) in a longitudinal cohort study.^26^ Consistent with these previous studies, the present study found significantly higher levels of EDN according to asthma control status. Furthermore, as expected from the function of EDN, we found negative correlations between lung function parameters (FEV_1_% and FEV_1_/FVC) and EDN levels. The present study aimed to investigate these associations according to specific asthma phenotype, comparing between AERD and ATA patients. LTE_4_ overproduction and type 2/eosinophilic airway inflammation are a major inflammatory pathway to present the phenotype of AERD. As early steps of this mechanism, epithelium‐derived cytokines, such as thymic stromal lymphopoietin, interleukin‐25, and interleukin‐33, activate mast cells and eosinophils and then leads to release cysteinyl leukotrienes.^27^, ^28^ Urinary LTE_4_ has been extensively studied as a reproducible biomarker determining the phenotype of AERD and reported to be elevated in patients with uncontrolled AERD.^29^, ^30^ Elevated levels of PEC or FeNO are also known for poor asthma control and risk factor for AE.^1^, ^11^, ^31^ The present study demonstrated that EDN levels (plasma, urine, and sputum) are the most important factor predicting UA in patients with AERD, but not in those with ATA. EDN was significantly correlated with PEC and LTE_4_. In the multivariate analysis sputum EDN remained as the only significant factor for poor asthma control in AERD patients. These results were consistently found, regardless of the outcome parameter used for the assessment of asthma control status including ACT, ACQ, or AQLQ scores. Several kinds of specimens have been used to analyze the levels of eosinophil degranulation products in asthma; however, there is no definite consensus on which sample is the most suitable to predict asthma control status. In the present study, EDN levels in sputum samples showed better predictability for UA, suggesting that EDN levels found in airway secretions (target tissues of asthma) may better predict airway inflammation and clinical outcome (asthma control status). The potential clinical implication of this observation warrants further studies.

Meanwhile, we could not find statistical significance of PEC, SEC, or FeNO levels for predicting poor asthma control in the multivariate analysis. This is in agreement with the previous studies that showed better performance of EDN level to assess asthma control status than PEC.^24^, ^26^ These findings collectively suggest that eosinophil activity may be a pivotal factor inducing poor asthma control rather than the level of PEC itself in patients with AERD.

As AERD is not fully understood by enhanced production of type 2 cytokines or cysteinyl leukotrienes, novel molecules related to activated eosinophils have been proposed to be implicated in AERD pathogenesis.^32^ Eosinophil extracellular trap (EET) which is released from activated eosinophils contains a mixture of web‐like DNA fibers and granule proteins.^33^ EET‐forming eosinophil counts were positively correlated with serum EDN levels and negatively correlated with FEV_1_% in patients with SA.^34^ In this context, EET and EDN have been postulated to be involved in the mechanism of activated eosinophils and potential therapeutic target for AERD.^32^, ^35^ Proteins, such as Rab proteins and vesicle‐associated membrane proteins, that regulate the degranulation process and release of granules (e.g., EDN, ECP, EPO, and MBP) from immune cells, were reported to have an important role in airway inflammation and hyperresponsiveness in vitro and in vivo.^36^, ^37^ Despite similar eosinophil count, Rab27a‐deficient mice crossed with interleukin‐5‐overexpressing mice showed significantly reduced levels of EPO in bronchoalveolar lavage.^37^ These studies have shown that eosinophil degranulation processes are critical for the progression and severity of type 2 airway inflammation. Consistent with this experimental study data, the present study also demonstrated that clinical outcome (asthma control status) was more closely related with the amount of degranulated product (EDN) than with eosinophil count itself. Therefore, the level of eosinophil degranulation product (EDN) may be a useful biomarker for predicting asthma control status in patients with AERD, even in patients on maintenance medication.

In addition, specific polymorphisms in the *RAB1A* gene were associated with the risk of AERD and a greater decline in FEV_1_% after aspirin challenges.^38^ A recent transcriptomic study also identified degranulation‐related genes, including *STX2*, and *RAB3B,* were up‐regulated in patients with AERD. These genetic studies have demonstrated that degranulation‐related genes are strongly associated with the risk of AERD. These findings support significant correlations between the EDN levels and asthma control status/the degree of airflow limitation in AERD patients, not in ATA patients. In addition, when the predictability of EDN levels for UA was compared between the AERD and ATA groups, good predictability was noted in the AERD group. These findings may be explained by previous genetic studies on close associations between degranulation‐related genes and the risk of AERD. Collectively, it is suggested that EDN may be closely associated with a specific phenotype of asthma and serves as a good phenotypic biomarker of UA in patients with AERD.

This is a cross‐sectional study; therefore, further studies with a longitudinal follow‐up study design are needed to assess the change and variability of EDN levels in AERD patients. The number of study subjects enrolled in the present study is small. Further studies with a larger sample size are needed to completely evaluate associations between EDN levels and asthma control status. Previous studies have implicated that genes associated with degranulation process may cause the increased levels of EDN in AERD patients with uncontrolled status; however, further functional studies on direct relationships among EDN, degranulation pathway, and clinical outcome are warranted in AERD patients.

In conclusion, the sputum EDN level may be a potential biomarker for identifying the asthma control status in patients with AERD, regardless of anti‐asthmatic medications.

## AUTHOR CONTRIBUTIONS

**Ga‐Young Ban**: data curation (lead); formal analysis (lead); investigation (equal); methodology (equal); project administration (equal); resources (equal); software (equal); validation (equal); visualization (lead); writing – original draft (lead). **Eun‐Mi Yang**: data curation (lead); formal analysis (equal); investigation (equal); methodology (lead); resources (lead); software (lead). **Young‐Min Ye**: conceptualization (equal); data curation (equal); project administration (equal); supervision (lead); writing – review and editing (lead). **Hae‐Sim Park**: conceptualization (equal); funding acquisition (lead); investigation (lead); methodology (lead); project administration (lead); supervision (lead); validation (equal); writing – review and editing (lead)

## CONFLICT OF INTEREST STATEMENT

The authors declare that they have no relevant conflicts of interest.

## Data Availability

Data available on request due to privacy/ethical restrictions.
